# Letter about the recently published Viewpoint by H. B. Rossiter and D. C. Poole

**DOI:** 10.1113/EP091805

**Published:** 2024-02-28

**Authors:** Maria Pia Francescato, Valentina Cettolo

**Affiliations:** ^1^ Department of Medicine University of Udine Udine Italy

We thank *Experimental Physiology* for publishing the Viewpoint (Rossiter & Poole, 2024) linked to our recently accepted paper (Francescato & Cettolo, 2024) suggesting that the issues illustrated therein deserve discussion. We want, however, to point out that the Viewpoint describes the ‘Independent‐breath’ (IND) algorithm with the following inaccuracies:

1. The ‘Independent‐breath’ algorithm is based on the pioneering work of Grønlund, not so the ‘Expiration‐only’ algorithm, as can be interpreted by reading the text (see first page of the Viewpoint, second column, lines 3–4).
2. In a previous work from our group, we stated that ‘the IND approach does not require any final off‐line optimization procedure, thus allowing real‐time calculations’ (Cettolo & Francescato, 2018; p. 1126, column 1, lines 7–8 of the Discussion) and that ‘These characteristics open the prospect to provide the investigators with the ability to assess the “true” alveolar gas exchange more accurately in real‐time’ (Cettolo & Francescato, 2018; p. 1128, column 2, lines 5–7). In the Viewpoint, however, it is stated that the algorithm (we guess the IND one) ‘can be applied only post hoc, once all the data have been collected’ (p. 1, column 2, lines 4–5).
3. For the IND algorithm, the start and end time points of each breath are actually identified on the ratio of fractional concentration of O_2_ and N_2_ (i.e., the $F_{O_{2}}$/$F_{N_{2}}$ ratio) or CO_2_ and N_2_ (i.e., the $F_{CO_{2}}$/$F_{N_{2}}$ ratio) at the end of two consecutive *expirations* (Cettolo & Francescato, 2018; p. 1122, column 2, line 7), not ‘in two consecutive *inspirations’*, as is reported in the Viewpoint (p. 2, column 1, line 28).
4. A by‐product of the real‐time calculations performed by the IND algorithm is that the breath‐specific reference ratio ($F_{O_{2}}$/$F_{N_{2}}$ or $F_{CO_{2}}$/$F_{N_{2}}$ ratio) can be found in all the breaths, thus avoiding that two consecutive breaths become one (Cettolo & Francescato, 2018; p. 1126, Section ‘The lack of contiguity in time of respiratory cycles: origin of the idea’ in the Discussion). Conversely, in the Viewpoint, it is stated that ‘…therefore two breaths become one under the more precise and accurate independent‐breath approach.’ (p. 2, column 1, end of paragraph 2). Notably, the name given to the IND algorithm derives from the independent calculation of gas exchange for each breath.

It should be remembered here that the Auchincloss’ gas exchange algorithm as well as its variants, that maintain the traditional timings of ‘a breath’ (Auchincloss et al., 1966; Swanson & Sherrill, 1983; Wessel et al., 1979), and Grønlund's algorithm along with the derived ones, that change the timings of ‘a breath’ (Capelli et al., 2001; Cettolo & Francescato, 2018; Grønlund, 1984), were all designed to estimate the gas exchange at the alveolar‐to‐capillary membrane. In our opinion, for exercise testing, the gas exchange values calculated using these algorithms cannot be directly compared with the ventilatory variables assessed at the mouth level, and commonly obtained analysing the flow signal for only the expiratory phase (e.g., ventilation, tidal volume). Indeed, the assumptions made at the mouth might not remain adequate for the alveolar level and thus, to be congruent, even all the ventilatory variables should be redefined. In the alveoli, the respiratory phenomena (e.g., air flow) likely occur with very smooth oscillations, similarly to what happens in the cardiocirculatory system, where the fluctuations in blood pressure can no longer be detected at the capillary level. Consequently, we believe that the redefinition of ‘a breath’ is more complex than changing only its timings, although this might seem the main challenge.

## AUTHOR CONTRIBUTIONS

Valentina Cettolo and Maria Pia Francescato have contributed equally to this work. Both authors have read and approved the final version of this manuscript and agree to be accountable for all aspects of the work in ensuring that questions related to the accuracy or integrity of any part of the work are appropriately investigated and resolved. All persons designated as authors qualify for authorship, and all those who qualify for authorship are listed.

## CONFLICT OF INTEREST

No competing interests declared.

## FUNDING INFORMATION

No funding was received for this work.
